# Perceptions and factors affecting the adoption of digital games for engineering education: a mixed-method research

**DOI:** 10.1186/s41239-022-00369-z

**Published:** 2023-01-07

**Authors:** Chioma Udeozor, Fernando Russo-Abegão, Jarka Glassey

**Affiliations:** Merz Court, School of Engineering, Newcastle University, Newcastle Upon Tyne, NE1 7RU UK

**Keywords:** Perceptions, Game-based learning, Engineering education, Mixed method research, UTAUT2

## Abstract

Digital games are considered relevant in higher education due to their ability to foster authentic, active and experiential learning opportunities that are of importance in engineering education. However, as a relatively new pedagogical tool, there is the need to understand the perceptions of engineering students as well as to identify factors that influence their adoption of games for learning. So far, only a few studies have investigated the perceptions of higher education students towards learning games and even fewer for engineering students. To bridge this research gap, the current study utilises a mixed-method research design to identify factors that influence the adoption of digital learning games by engineering students as well as their overall perceptions of the use of games for engineering education. Results from the analysed quantitative and qualitative data suggest that engineering students value fun and engagement as well as relevance to the curriculum as factors that would influence their intentions to use digital games for engineering education. Students also showed openness to the use of digital games for learning, but resistance to their use for assessment. These findings have implications for the design of games and classroom deployment of games, as these provide insights to game designers and educators on the factors to consider in the design and classroom deployment of games, respectively.

## Introduction

In higher education institutions (HEI), the growing need to improve the learning experiences of students and support the development of skills and knowledge required by current and future employers is driving investments in innovative technologies. Digital games and other immersive technologies have gained considerable attention as educational and training tools that provide realistic and experiential learning environments. The recent rapid increase in interest in these technologies has been lately accelerated by the limitations to classroom teaching and learning, and the challenges posed by the Covid-19 pandemic restrictions. Many HEIs are investing in simulation games as well as virtual and augmented realities for laboratory and practical training (Glassey & Magalhães, 2020). Although there have been limited reports of the use of games in the engineering domain compared to other domains, an increasing number of studies are now being reported (Gordillo et al., 2022; Solmaz & Van Gerven, 2021; Udeozor et al., 2022), with positive outcomes reported from using these for education and training (e.g. Suescún et al., 2018; Xenos & Velli, 2020). As with any new technology, it is believed that understanding the perceptions of users enhances the adoption and intended outcomes of the technology. So far, many studies have investigated the perceptions of students and factors affecting their adoption of games for learning (Pando-Garcia et al., 2016; Ramírez-Correa et al., 2019; Saleh et al., 2014). However, there are limited studies exploring perceptions towards games from the viewpoint of engineering students. The current study, therefore, evaluates the perceptions of engineering students towards digital games and the factors that affect their adoption of digital games for education.

Digital games are known to offer a lot of educational benefits. Proponents of digital game-based learning believe that when used for learning purposes, digital games could improve the learning experiences and the outcomes of students (Kazimoglu, 2020; Plass et al., 2015). Games are known to be intrinsically motivating and engaging (Garris et al., 2002). As an educational tool, digital games are considered relevant in HEIs due to their ability to foster authentic, active and experiential learning opportunities (Connolly et al., 2012; Whitton, 2009). As the role of engineers involves the design, development and improvement of products and processes, and the education of engineering students is limited by practical real-world experiences (Johnson & Singh, 2019), digital games have the potential to bridge the knowledge and skills gap. With the ability to simulate real-life environments that are interactive and offer immediate feedback, engineering students can gain practical industry-relevant skills without being limited by access to the industrial environment. In addition to technical engineering skills, the immersive and engaging characteristics of games also provide an opportunity to enhance the development of twenty-first century transferrable skills such as collaboration and problem-solving skills required by employers (Bahadoorsingh et al., 2016).

The use of games for engineering education has been found to offer similar, and sometimes better outcomes when compared to conventional teaching methods. Suescún et al. (2018) found comparable test performance among two groups of students: a game group that used games for learning, and a non-game group that used conventional pedagogical tools. When used for ethics education, Xenos and Velli (2020) noted a significant improvement in post-test scores of students after learning through games compared to their pre-game test scores. Other studies like those of Dib and Adamo-Villani (2014); Flores et al. (2020); and Perini et al. (2018) also found a significant improvement in learning outcomes with the use of digital games. Notwithstanding these positive outcomes, the adoption and the use of digital games for engineering education are limited when compared to domains like medical and business education.

As a professional discipline requiring certain professional competencies that are often contextual to the work environment, engineering education can benefit from the use of digital games to complement current educational practices. Digital games have proven to be effective in simulating realistic environments, enhancing motivation and learning performance (Garris et al., 2002; Kazimoglu, 2020; Plass et al., 2015). When applied to engineering education, digital games could enhance the professional skills of graduates and potentially produce job-ready employees for the chemical industry. To enhance the adoption of games or any new technology and ensure intended outcomes, it is believed that understanding the perceptions of students is of great importance (Beavi et al., 2015). A few studies have explored the views of students toward game-based learning with the outcomes of most of these being generally positive (Andreu-Andrés & García-Casas, 2011; Sevim-Cirak & Yıldırım, 2020; Thanasi-Boçe, 2020; Yue & Tze, 2015). In the study by Bolliger et al. (2015), 81% of the participants agreed that games offer opportunities to experiment with knowledge. Fifty-eight per cent (58%) of the participants in another study agreed or strongly agreed with statements measuring their views on the efficacy of digital games to improve learning. Most recently, the outcomes of another study showed that engineering students have positive sentiments toward using digital games for education (Udeozor et al., 2021, 2022). Although these studies show that students are open to the idea of using games for learning, fewer studies have explored factors that affect their adoption of digital games for education. Using conceptual models like the Technology Acceptance Model (TAM) and the Unified Theory of Acceptance and Use of Technology (UTAUT), some studies identified ‘perceived ease of use’ and ‘perceived usefulness’ (Bourgonjon et al., 2010; Fagan et al., 2012), and ‘enjoyment’ (Beavis et al., 2015) as factors influencing the adoption of games for learning. However, the majority of these studies involved primary and secondary students whose outlook toward the subject of game-based learning may differ completely from those of higher education students, particularly students in engineering disciplines.

Understanding the perceptions of students and factors that influence their adoption of digital games for engineering education is crucial to inform the development of effective games. It will also enable educators to utilise appropriate instructional designs based on the needs of students when implementing digital games for engineering education. This study utilises mixed-method research to identify factors that would influence the use of digital games by engineering students as well as to evaluate their perceptions of game-based learning for engineering education. It builds upon the outcomes of a previous study that explored the perceptions of engineering students and professionals (Udeozor et al., 2021). The current study provides solid evidence of the views of engineering students with a larger sample size that allowed for more sophisticated statistical analysis that identifies factors that have the strongest influence on the use of educational games by engineering students. Following the sequential explanatory design (Creswell, 2011), quantitative and qualitative data were collected and analysed to provide a holistic understanding of the opinions of engineering students about learning games. So far, to the best knowledge of the authors, only a few studies have examined the perceptions of engineering students from a quantitative viewpoint (e.g. Andreu-Andrés & García-Casas, 2011). Additionally, concerns have been raised about the effectiveness of existing technology acceptance models commonly adopted for games-related studies (Hsu & Lu, 2004). The current study, therefore, begins to answer the question of what factors affect the use of digital games by higher education students using both qualitative and quantitative data. The results of this mixed-method study will help inform the design of more appropriate technology acceptance models relevant to game-based learning.

### Theoretical framework and hypotheses generation

#### The extended unified theory of acceptance and use of technology (UTAUT2)

To identify factors that influence the adoption of digital games for engineering education, the extended Unified Theory of Acceptance and Use of Technology (UTAUT2) model was used. The UTAUT2 framework (Venkatesh et al., 2012) is an extended version of the UTAUT model. The original UTAUT model was developed by Venkatesh et al. (2003) taking into account seven prominent models/theories: Technology Acceptance Model (TAM), Theory of planned behaviour, Theory of reasoned action, Motivational model, Model of PC utilisation, Innovation diffusion theory, and Social cognitive theory. In 2012, Venkatesh and colleagues extended the UTAUT by incorporating three new constructs: hedonic motivation, price value and habit, to form the UTAUT2 model (Venkatesh et al., 2012). Compared to UTAUT, UTAUT2 has been reported to be able to explain up to 74% (56% for UTAUT) of variance in the behavioural intentions to use a new technology, and 52% (40% for UTAUT) of the variance in technology use behaviour (Venkatesh et al., 2016; Wang et al., 2020). The UTAUT2 was therefore chosen for this study as it offers better predictive power and has been widely used in related research (Kumar & Bervell, 2019; Ramírez-Correa et al., 2019; Samsudeen & Mohamed, 2019; Toyoda et al., 2020; Wang et al., 2020).

**Fig. 1:**
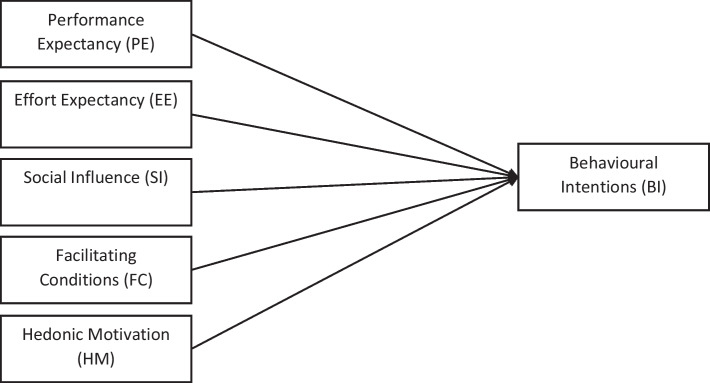
The modified UTAUT2 model

The UTAUT2 theorizes that Performance Expectancy (PE), Effort Expectancy (EE), Social Influence (SI), Facilitating Conditions (FC), Hedonic Motivation (HM), Price Value (PV) and Habit (H) are direct determinants of the intention of an individual (Behavioural Intentions, BI) to use technology, while FC, HM, PV and H are direct determinants of usage. As shown in Fig. 1, a modified version of the UTAUT2 was used in this study to determine the factors that influence the behavioural intentions of engineering students to use digital learning games.

*Performance Expectancy (PE)* is defined as the degree to which an individual believes that using a given technology will enable them to perform a certain activity. This variable represents the usefulness of usage of a technology, and it is often considered the strongest predictor of behavioural intention to use a new technology (Venkatesh et al., 2003). Digital games have the capacity to improve the learning experiences of students making them learn better and perform better when assessed, hence it is expected that the hypothesis H1 is formulated:

H1: Performance Expectancy will have a significant influence on the intentions of students to use digital games for learning.

*Effort Expectancy (EE*) is defined as the perception of the degree of ease associated with the use of a technology. Like Performance Expectancy, Effort Expectancy is often found to be a significant determinant of behavioural intention to use a technology (Fagan et al., 2012; Venkatesh et al., 2003). Therefore, it is hypothesised that:

H2: Effort Expectancy will have a significant influence on the intentions of students to use digital games for learning.

*Social Influence (SI)* is defined as the extent to which an individual perceives that people who are important to them believe that they should use a new technology (Venkatesh et al., 2003). With regards to the use of digital games for engineering education, SI can be viewed as the effect of the social environment, for instance, teachers, peers, families and friends on the behavioural intentions of students to use digital learning games. Hence, it is predicted that:

H3: Social Influence will have a significant influence on the intentions of students to use digital games for learning.

*Facilitating Conditions (FC)* refers to the extent to which one believes that appropriate infrastructure exists to enhance the use of a technology (Venkatesh et al., 2003)*.* This can be viewed as elements or factors that enhance or hinder the adoption of a new technology. In the case of learning games, this could include the support, training or skills needed to navigate the game. It is therefore expected that:

H4: Facilitating Conditions will have a significant influence on the intentions of students to use digital games for learning.

*Hedonic Motivation (HM)* is referred to as the fun or pleasure one derives from using a technology (Venkatesh et al., 2012). HM is conceptualised as perceived enjoyment and has been found to have the greatest influence on intentions to play games (Ha et al., 2007). Digital games are generally intrinsically motivating because of the elements of fun and engagement associated with them. It is therefore expected that this factor will play a major role in the intentions of students to use digital learning games. Hence, from hypothesis H5 it follows that:

H5: Hedonic Motivation will have a significant influence on the intentions of students to use digital games for learning.

*Behavioural Intentions (BI)* describe the intentions, likelihood or probability that an individual will use a new technology (Venkatesh et al., 2012). BI plays a major role in determining the adoption and actual use of a new technology. Nevertheless, this study does not attempt to predict nor explain the actual use of games by students but aims at explaining factors that affect their behavioural intentions to use games for engineering education.

## Methodology

**Fig. 2:**
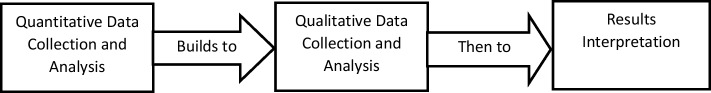
Explanatory sequential design (adopted from Creswell, 2011)

This mixed-method research follows the explanatory sequential design (Creswell, 2011), which involves the collection of quantitative data, followed by qualitative data, as shown in Fig. 2. This design method allows for in-depth exploration of findings from quantitative data through qualitative data collection. It also provides the opportunity for the elaboration of opinions that may not have been possible with only quantitative data collection. For this study, an online questionnaire and a focus group interview were used for quantitative and qualitative data collections, respectively.

## Quantitative study

### Methods

#### Participants

125 chemical engineering students from three European universities—Newcastle University, KU Leuven and Imperial College, London took part in this study. Convenience sampling method was used to recruit these participants (Creswell, 2011). As shown in Table 1, there were 70% male and 30% female students with varying gameplay experiences.

**Table 1:** Demographic data of participants, n = 125

		Absolute frequency	Percentage
Gender	Male	87	69.6
	Female	37	29.6
	Unspecified	1	0.8
Age	Under 20 years old	50	40.0
	20–29 years old	74	59.2
	30–39 years old	1	0.8
Prior gaming experience	Yes	117	93.6
	No	8	6.4
Gaming habits (hours)	Less than 5 h	54	43.2
	5–10 h	41	32.8
	11–20 h	17	13.6
	21–30 h	7	5.6
	31 h or more	1	0.8
	Unspecified	5	4.0
Game enjoyment	Low (1–4)	14	11.2
	Average (5–7)	49	39.2
	High(8–10)	58	46.4
	Unspecified	4	3.2

#### Questionnaire design

To identify the factors that influence the intentions of students to use digital games for engineering education, an online survey questionnaire was utilised. The questionnaire collected demographic data of students, their game experiences and finally the perceptions of students measured on 6 constructs based on the UTAUT2 model (see Appendix 1). The questionnaire adapted from the UTAUT2 model was chosen for its wide adoption and validation as a technology acceptance model (Kumar & Bervell, 2019; Ramírez-Correa et al., 2019; Toyoda et al., 2020). The wording of each question was modified to suit the study context. The constructs here were measured on a 6-point Likert scale, with 1 used for ‘Strongly Disagree’ and 6 for ‘Strongly Agree’. A 6-point Likert scale was chosen as it is believed to produce a higher discrimination and reliability trend than a 5-point scale (Chomeya, 2010). Before using the questionnaire for data collection, validity checks were carried out by academic experts and volunteer postgraduate chemical engineering students. Based on feedback, changes were made to the wording of the questions where needed.

#### Data collection and analysis

Data were collected from the student participants on three different occasions. For all three data collection events, students also took part in gameplay sessions. First, participants were requested to complete the online questionnaire to evaluate their perceptions of learning games for engineering education before proceeding to the gameplay sessions. Before utilising this questionnaire for data collection, ethical approval was obtained from the Ethics Committee at Newcastle University. As shown in Table 1, a total of 125 students completed this questionnaire.

### Results

An exploratory partial least square Structural Equation Modelling analysis (SEM-PLS) was used to determine the predictors of the behavioural intentions of engineering students to use learning games based on the modified UTAUT2 model. To do this, measurement model and structural model analyses were conducted (Hair et al., 2017) using the SmartPLS™ Version 3 software. To determine the minimum sample size for these analyses, Cohen’s statistical power analysis for multiple regression models was carried out (Cohen, 1992; Nitzl, 2016). This minimum sample size estimation method is considered more rigorous and appropriate for determining the minimum sample size for SEM-PLS analysis compared to the 10 times rule (Hair et al., 2021; Kock & Hadaya, 2018). Based on this approach, the minimum sample size for our analyses is 92 given 5 independent variables, and working with a statistical power of 0.8, a significance level of 0.05 and a medium effect size (Cohen, 1992; Nitzl, 2016). Therefore, a sample size of 125 used in this study is sufficient for the current analyses.

#### Measurement model

The measurement model aimed to evaluate the reliability and validity of the constructs used. As shown in Table 2, the Cronbach’s alpha and composite reliability (CR) scores, which are measures of internal consistency reliability of the measurement constructs, were all above the 0.6 minimum scores (Hair et al., 2017). This shows that the measurement constructs have strong internal consistency reliability indicating good correlations between items intended to measure the same constructs.

**Table 2:** Reliability and validity measures

Constructs	Item codes	Mean score	Standard deviation	Factor Loading	AVE^a^	Cronbach’s Alpha	CR^a^
Performance expectancy (PE)	PE_1	4.36	0.90	0.864	0.681	0.847	0.895
	PE_2	4.67	0.84	0.794			
	PE_3	4.50	0.90	0.802			
	PE_4	4.53	0.96	0.839			
Effort expectancy (EE)	*EE_1				0.764	0.845	0.906
	EE_2	4.62	0.94	0.81			
	EE_3	4.51	0.91	0.896			
	EE_4	4.67	0.95	0.913			
Social influence (SI)	SI_1	4.63	1.03	0.724	0.674	0.784	0.86
	SI_2	4.30	1.03	0.892			
	SI_3	4.76	0.95	0.838			
Facilitating condition (FC)	*FC_1				0.757	0.707	0.861
	FC_2	4.52	1.14	0.785			
	FC_3	3.97	1.31	0.948			
Hedonic motivation (HM)	HM_1	4.02	0.99	0.936	0.878	0.931	0.956
	HM_2	4.18	0.94	0.947			
	HM_3	3.96	1.07	0.928			
Behavioural intentions (BI)	BI_1	3.40	1.15	0.888	0.753	0.836	0.901
	BI_2	3.64	1.17	0.894			
	BI_3	4.26	1.22	0.819			

*Removed due to low outer loading (< 0.7); ^a^Average Variance Extracted; ^b^Composite Reliability; Item codes represent individual questions associated with a given constructs

Furthermore, the validity of the constructs was determined by evaluating the convergent and discriminant validities as recommended by Hair et al. (2017). As shown in Table 2, the factor loadings for each survey question/item and the average variance extracted for each construct (AVE) were greater than the recommended minimum of 0.708 and 0.5, respectively. Additionally, the Heterotrait-Monotrait ratio (HTMT), a measure of discriminant validity, shown in Table 3, was found to be below the 0.9 recommended maximum value (Gold et al., 2001). These, therefore, indicate that the discriminant and convergent validity conditions of our model were met, hence the constructs and items can be said to be both valid and reliable.

**Table 3:** Heterotrait-Monotrait ratio (HTMT) measure of discriminant validity

	BI	EE	FC	HM	PE	SI
Behavioural intentions (BI)						
Effort expectancy (EE)	0.357					
Facilitating conditions (FC)	0.548	0.352				
Hedonic motivation (HM)	0.764	0.331	0.493			
Performance expectancy (PE)	0.574	0.360	0.781	0.577		
Social influence (SI)	0.413	0.494	0.408	0.451	0.593	

#### Structural model

**Fig. 3:**
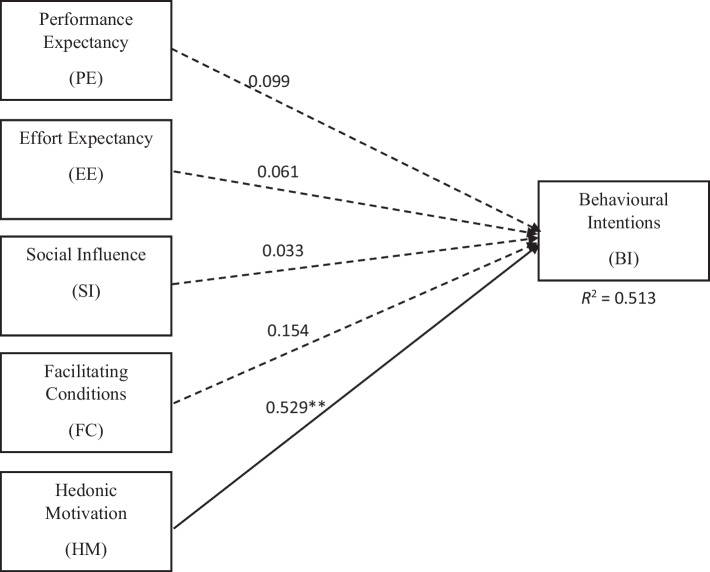
Structural equation model to predict factors that influence intentions of engineering students to use digital games for engineering education (**p < 0.001)

Since the validity and reliability of the constructs were established with the measurement model, the next step was to perform the structural model assessment. This involved determining the coefficient of determination (*R*^*2*^) and the significance of path coefficients (*B*) (Hair et al., 2017). Before proceeding with these analyses, the model was assessed to ensure there are no collinearity issues (Hair et al., 2017). It was found that all constructs had variance inflation factor (VIF) values of less than 3, which is the recommended threshold, indicating that there were no multicollinearity issues. To evaluate the relationships between the constructs, the path coefficients and their influence on the model were analysed as shown in Fig. 3.

From Fig. 3, the variance explained by PE, EE, SI, FC and HM for behavioural intentions of students to adopt digital games for learning were 0.099, 0.061, 0.033, 0.154, and 0.529, respectively. From this result, it was found that only HM had a significant positive influence on the behavioural intentions of students to use games for engineering education, supporting H5. Therefore, the hypotheses H1, H2, H3, and H4, which stated that PE, EE, SI, and FC, respectively, have a significant influence on the behavioural intentions of students to adopt digital games for learning, are rejected. Given that *R*^*2*^ > 0.2, the adapted UTAUT2 model is considered acceptable for this behavioural study (Hair et al., 2017) as it is able to accurately predict 51.3% variance in the BI of engineering students to use digital games for learning.

## Qualitative study

### Methods

The goal of this second phase of the current study was to understand the perceptions of students towards digital learning games for engineering education, explore further the factors that affect their use of digital games as well as collect additional information that might not have been captured by the questionnaire. The questions asked during the interview centred on three main areas:

RQ1: What are the views of students on the use of games for engineering education?

RQ2: What attributes do students expect from a digital game for engineering education?

RQ2: What are the views of students towards games for assessments?

#### Participants

Seven volunteers who took part in the quantitative study participated in the focus group interview. The participants were seven chemical engineering students from Imperial College, London. There were four female and three male student participants.

#### Data collection and analysis

A one-hour focus group interview was conducted with all seven participants. A semi-structured interview approach was used (Creswell, 2011). Due to the COVID-19 pandemic restrictions, the interview took place remotely via Microsoft Teams. With the permission of the participants and appropriate ethical approval received from the ethical board at Newcastle University, the interview was recorded. Both video recordings and transcripts were obtained. A systematic approach was used for the analysis of the qualitative data. First, the automatically generated transcripts were carefully read through, compared against the video recording and edited where necessary for accuracy. Next, responses to each question were extracted. To identify the main points from the discussion, the responses to each question were read and key points (codes) highlighted. This process was manually carried out in Microsoft Word because of the low number of participants involved. With key codes highlighted, points that convey similar ideas were grouped to form themes as shown in Table 4.

**Table 4:** Matrix for coded interview questions, n = 7

Research questions	Number of participants	Codes	Themes
Views towards games for engineering education	5	Effective for learning Enhance visualisation Good for skills learning Experiential learning Project-based learning	Positive
	2	Not better than existing pedagogies Faster to learn the existing way	Negative
Qualities expected of engineering educational games	3	Collaboration Competition Leaderboard	Motivation/Engagement
	3	Clear objectives Progressive learning	Relevance
	3	Visual/aesthetically pleasing More than just for learning	Quality design
Views on games for assessment	7	Changes exam revision strategies Extra stress	Test anxiety
		Poor gaming skills might affect grades	Gaming skills interference
		Glitches and poor connectivity	Technical concerns
	1	Depends on how it is introduced	Implementation practicalities

## Results

### RQ1: views of students towards using digital games for engineering education.

#### Theme 1: positive

When asked if digital games could be used for engineering education, five students (71%) agreed to some degree that digital games are suitable for engineering education. Most of the participants were of the opinion that games could be used to learn, but for engineering education, the opportunities were thought to be limited to some modules:

‘I think you could learn something entirely through games…’

‘I reckon, something like heat transfer could be learned entirely through gaming…

…visualise a lot better.’

‘… I think for things like projects (would) work with games... so more (useful) for project work (knowledge application) versus learning content (knowledge acquisition).’

#### Theme 2: negative

Two participants did not think that games were suitable for engineering education. They believed that games could not be useful for every engineering subject and that it would be practically difficult to replicate engineering systems in games:

‘…there’s nothing that you can learn through a game that you cannot learn by just reading some [pages] in a short amount’

‘…because of how complex real systems (in engineering) are it’s often really hard to mimic those in a game setting.’

Additionally, all of the seven participants expressed the view that games should be used as supplements or add-ons rather than as stand-alone pedagogical tools. These responses show that, although the majority of the students perceive games to be useful for engineering education, there are some concerns about their suitability and practical use.

### RQ2: qualities expected from engineering educational games

Participants were also asked to describe qualities that would positively influence their adoption and use of digital games. The themes that emerged from the responses are:

#### Theme 1: motivation/engagement

This was the most frequently occurring theme in the data. This aligns with the observations in the quantitative phase of this study, in which hedonic motivation was found to be the most significant factor for game adoption. Participants mentioned some key factors that would enhance their gameplay experience and influence their adoption of games for learning. Features of collaboration and competition were mentioned by these students as qualities they would appreciate in educational games:

‘…Say that aspect of competitiveness is always helpful in sense…, being able to play against other people.

Always helps to like make it more interesting, because then it’s not just us, (it’s) you and your friends, seeing how you will do in comparison to others…’

‘…Important playing with someone else makes a game infinitely more fun…’

#### Theme 2: relevance

Participants expressed the need for a game to be relevant to their learning journey. They expect to see a clear link between learning objectives and the proposed game objectives. Relevance here can be conceptualised as the perceived usefulness, which in the UTAUT2 model is known as Performance Expectancy (PE):

‘…So I think you should make it clear what exactly you're teaching and not (just an) educational game because if someone's like, trying to revise through a game…’

‘…you (should) learn something new (with the game)…’

‘…I feel like the really good games have an objective like Mario international game you saved princess. You know, like, really clear cut.

For an educational game, (it should say) ‘You need to do this at the end of the time… Your outcomes will be, you will have completed the learning objective x y …’

#### Theme 3: quality game design

The last theme that emerged from the data was ‘quality game design’. Participants mentioned the need for an educational game to be aesthetically pleasing and do more than just teach. For these students, games should be well designed and games should do more than just replicate what students can learn from existing pedagogical tools:

‘…like the visualisation will have to be good, done by someone who knows some software…, it'll have to be together and feel like a complete kind of game.’

‘… (What)I’d like to say is that even educational game, setting education as the objective, might be a tad boring…

…goal itself should be different because if the end goal is education, why am I playing a game? I could just read that (from books) to also achieve that end goal.’

Another student elaborated on the need for a good educational game to provide instant and continuous feedback. This student also mentioned the need for a leader board that should tell players how well they are doing against pre-set competencies.

Having heard the views of the students regarding the qualities they expect from an educational game for engineering education, next, students were asked to talk about engineering modules/courses that would benefit from game-based learning. Modules that generally require students to operate or design processes, and/or products, were described as those that would most likely improve the learning experience. As the participants of this study were chemical engineering students, the following chemical engineering modules were mentioned: Fluid Mechanics, Heat and Mass Transfer, Safety, Separation Processes, Reaction Engineering, and Design Processes.

### RQ3: views of students towards games for assessments

Participants were asked if they would be happy to be assessed with games. Unanimously, they said ‘no’. When probed to give reasons for their answer, they gave several reasons which are described in the themes below.

#### Theme 1: test anxiety

The common point raised by many of the participants was that assessment with games would create an extra layer of stress and anxiety. They reiterated that traditional assessments already cause some levels of stress to students and that changing the format of the test would not make things any easier. Additionally, it was said that changing to game-based assessment would require changing the way students study and prepare for exams:

‘...I wouldn’t, I would never really want to have games replace my exams, just because I’m used to like revising a certain way for an exam and knowing kind of what to expect. So, even now, like you saw how like online exams. People were panicking and that’s not even changing…’

‘…But yeah, I think just having the idea of a game as an assessment, adds a level of extra stress that we don’t really like. I love games, but I still wouldn’t want to do a game as an assessment…’

#### Theme 2: gaming skills interference

In addition to the perceived stress students anticipate with game-based assessment, the effect of gaming skills or competencies on performance was also raised. Participants worried that performance in game-based assessments would be influenced by gaming skills of the students:

‘…but I think the (game-based) examination requires another skill that you have to be good at, in addition to everything you’re being tested on…

As I play like much less than other people and then when I do occasionally play games, I’m like way worse. It is a disadvantage…’

#### Theme 3: technical concerns

One participant elaborated on the technical uncertainties associated with the use of digital games for something as important as an assessment. They described the kind of technical issues that arise during gameplay and how this could adversely affect students:

‘…I still wouldn’t want to do a game as an assessment because I know what can go wrong in games. I somehow broke the game we were playing twice by mistake. So just because when it comes to things like computers, especially like a lot of games. There’s bugs and sometimes they're not found by even the triple A companies upon release. So if that comes into play during an exam, if something bugs, that’s an entire level of stress that I don’t want to deal with…’

#### Theme 4: implementation practicalities

Although generally, the participants would prefer not to be assessed with games, some of them showed some flexibility. Some students were open to the use of games for assessment only if they had little or no impact on their grades. One student said they would not mind being assessed with games if it accounted for no more than 5% of the module score:

‘… (I) do not want to do it as an official exam. I will enjoy it if it was not timed and if it was like worth 5% of my module…’

Another student would consider game-based assessment only if it was gradually rolled out throughout a semester and not just once at the end of the module:

‘…I think I’d be open to it completely dependent on how it was implemented. So for example, if at the start of the module, the teacher was like hey I've got a new thing I want to try out this year. I want to try out again at the final exam and we're going to go through a few examples in the class of how a game-based exam might work… However, if a person just came in and slapped me down with a game and was like, plug this into your computer. This is going to be your assessment. I think I would crap myself like it’s just not it…’

## Discussion and conclusion

This research aimed to identify factors that influence the behavioural intentions of engineering students to use games for learning as well as their perceptions of digital games used for engineering education. This research takes further a previous study that compared the perceptions of chemical engineering students and professionals (Udeozor et al., 2021) by diving deeper to understand factors influencing the use of games by engineering students using data from a larger sample size. Following mixed-method research, the results of the quantitative data analysis indicate that of all considered constructs, only hedonic motivation (HM) had a statistically significant influence on the intentions of students to adopt digital games for engineering education. This suggests that the cohorts of students who took part in this study consider pleasure, fun and enjoyment derived from gameplay as the most important factor that would determine their adoption of digital games for engineering education. This aligns with the findings of others looking at perceptions toward games in the higher education context (Andreu-Andrés & García-Casas, 2011; Duffull & Peterson, 2020; Udeozor et al., 2021; Wang et al., 2020). Similar to the current finding, Wang and colleagues found HM to be the singular and most significant factor that influenced the behavioural intentions of management students to use business games for learning. Performance Expectancy (PE), Effort Expectancy (EE) and Social Influence (SI) did not have any significant influence on the intentions of students to use business games. Using a questionnaire with open-ended questions, Duffull and Peterson (2020) found that one of the emerging themes from the responses of pharmacy students regarding games use for learning was ‘fun’. They found that fun was an important factor for repeated gameplay and immersion when adopting games for learning. Although unexpected, it is not completely surprising that only HM had a significant influence on behavioural intentions in the current study. With the cohort of students in this study being undergraduates in their early 20 s and generally regarded as digital natives (Prensky, 2001), it is understandable that they consider fun, enjoyment and pleasure derived from gameplay as important factors that will determine their adoption of games for learning purposes. A contrast can be found in the study of Toyoda et al. (2020) which reported that for professional chemical engineers, PE had the most significant influence on their intentions to use virtual reality for training purposes. This difference has been attributed to the value the different groups of users place on these technologies. While professionals are likely to adopt these technologies to become better at their jobs, students on the other hand would most likely adopt these because they are fun and engaging to learn with (Udeozor et al., 2021). Inconsistencies in results were also found in studies investigating perceptions of higher education students (Estriegana et al., 2019; Malaquias et al., 2018; Pando-Garcia et al., 2016; Zulfiqar et al., 2021). These studies found PE, EE and/or SI to have the most influence on the intentions of students to use games for learning. Inconsistencies and unexplainable findings in similar game-related studies using existing technology acceptance models such as UTAUT2 have been attributed to the likelihood that these models are possibly missing some important constructs necessary for measuring perceptions and acceptance of games for learning (Hsu & Lu, 2004). Therefore, in addition to quantitative data collection, the current study went further to collect qualitative data to gain a holistic understanding of the perceptions and factors influencing the adoption of digital games by engineering students.

The findings of the qualitative study showed that students are open to using games for learning and believe that digital games can be useful for engineering education. However, students emphasised that games be used as add-ons and not stand-alone pedagogical tools. There were also concerns about designing engineering games given the complexity of simulating engineering systems. When describing qualities that they would expect of digital games designed for learning, students mentioned the need for games to be collaborative and/or competitive games, particularly for higher education. They expect games to be relevant to their learning as well as fun, qualities that lend themselves to PE and HM, respectively. Although PE was not found to have a significant influence on the intentions of students to adopt games in the quantitative phase of this study, the majority of the participants clearly stated or implied that the goals of digital games for learning should clearly align with their curriculum learning outcomes as found in previous studies (Estriegana et al., 2019; Zulfiqar et al., 2021). Lastly, the design quality of games was mentioned as a factor that would affect the use of games for learning. Students expect educational games to be well designed and complete to be attractive enough for learning. A well-designed game was described as one that is aesthetically pleasing with instant and continuous feedback system.

Digital games provide opportunities for authentic assessments and offer many benefits such as the application of modern psychological theory to assessment, increasing assessment coherence and enabling the measurement of ‘hard-to-measure’ constructs that would otherwise be difficult to achieve (Buckley et al., 2021). Nonetheless, as a non-conventional assessment tool, it was necessary to hear what engineering students make of these. Although a majority of students in the focus group believe that games can be useful for engineering education and are willing to adopt them for learning, they did not share the same sentiments about the use of games for assessment as also found in the study by Cook-Chennault and Villanueva (2020). While a few students would consider using them for assessment depending on how they are rolled out and on the weighting on the overall grade, most of the students believe that using games for assessment would increase test anxiety. This is a particularly interesting finding considering that proponents of game-based assessments claim otherwise (Mavridis & Tsiatsos, 2017; Shute et al., 2017). Contrary to the findings of the current study, Mavridis and Tsiatsos (2017) found that test anxiety is lower with game-based assessments compared to traditional assessments and that students had positive attitudes toward game-based assessments. This inconsistency in findings could be due to the fact that in the study of Mavridis and Tsiatsos, students already experienced assessment through games and made judgements based on that, whereas, in the current study, students merely described their perceptions of game-based assessment with no prior experience. Furthermore, while students in the current study looked at game-based assessment from a high-stake assessment point of view, the result of the study of Mavridis and Tsiatsos was based on research with little or no implication on the grades of students. In addition to perceived test anxiety, students also mentioned gaming skills, technical issues and implementation practicalities as issues with using games for assessment.

In summary, aligned with the outcome of the preceding study, the elements of fun and engagement/motivation associated with hedonic motivation (HM) seemed to have the strongest impact on the intentions of students to use games for engineering education from both the quantitative and qualitative results. Whereas PE was not identified in the quantitative study as an influential factor, the qualitative data points to it as a factor of importance to engineering students. These findings from both the quantitative and qualitative phases of this study strengthen the argument for mixed-method research. The additional findings from the qualitative study provide an in-depth understanding of possible factors that could play crucial roles in enhancing the adoption of digital games by engineering as well as higher education students. These results could be valuable to digital game designers by providing insights into factors that should be considered when designing educational games. Despite the overall positive perceptions towards games for learning, the strong and negative opinions towards the use of games for assessment were attributed to reasons such as increased anxiety, changes to the old ways of studying and gaming skills interference. With careful consideration given to these concerns, it is recommended that games be gradually introduced in classrooms, initially as an additional learning tool. Sufficient time should be allowed before game-based assessments are implemented, particularly in summative assessments that contribute to the grades of students. This will allow students time to get comfortable with the new pedagogical tool and potentially prevent pushbacks and unintended negative effects on student performance.

### Limitations and future studies

This study benefits from the best of both quantitative and qualitative research, however, there are some limitations to the study which could inform future studies. First, while the sample size for the quantitative phase of this study is considered adequate, the sampling method used here limits the generalisability of the results. Future studies should repeat this study using probabilistic sampling strategies (Creswell, 2011) with larger sample sizes. Secondly, the qualitative study includes participants from a single university which also has implications for the generalisation of the findings. Future studies should consider including participants from a range of institutions to allow for findings to be generalisable to the broader population. Lastly, as indicated at the beginning of this study, concerns about the appropriateness of existing technology acceptance models such as the UTAUT2 used in this study for game-related research also raise questions about the results of the SEM-PLS analysis. With insights from the findings of this study, future research should focus on developing a game-based learning acceptance model appropriate for examining perceptions and identifying factors that play influential roles in the adoption of games by learners.

## Data Availability

The datasets used and/or analysed during the current study are available from the corresponding author on reasonable request.

## Appendix 1

Questionnaire item used in the study.

**Table:**

Measurement Constructs	Item codes	Items
Performance Expectancy	PE_1	I think that I would find digital games useful for learning engineering modules
	PE_2	I think that playing games designed to teach engineering concepts would increase my engineering knowledge
	PE_3	I think that playing educational games would enable me learn more quickly
	PE_4	Using digital games to learn engineering modules will increase the quality of my learning experience
Effort Expectancy	EE_1	I expect that my interaction with a well-designed engineering game would be clear and understandable
	EE_2	I expect that it would be easy for me to become skillful at the game
	EE_3	I expect to find games designed to teach engineering concepts easy to play
	EE_4	I expect that operating such games would be easy for me
Social Influence	SI_1	My teachers would expect me to use games for learning, if made available
	SI_2	My peers will be supportive of my use of games for learning
	SI_3	My teachers will be very supportive of my use of games for learning
Facilitating Condition	FC_1	I have the resources necessary to play games for learning purposes
	FC_2	My university will provide the necessary support for me to use games for learning
	FC_3	Playing digital games for learning fits well with the way I learn
Hedonic Motivation	HM_1	I will really enjoy playing games to learn
	HM_2	I think that playing engineering games will be fun
	HM_3	I think that playing engineering games will be very entertaining
Behavioural Intentions	BI_1	After the Recycling game, I intend to use games to learn again in the near future
	BI_2	I will continue to use games to learn engineering principles, if made available to me
	BI_3	I am open to using games to improve my knowledge of chemical engineering principles, if made available to me
